# Expression analysis of circ-AKT3 and circ-EGLN3 in BK polyomavirus-infected kidney transplant recipients: potential biomarkers for viral reactivation

**DOI:** 10.1186/s12879-025-11911-5

**Published:** 2025-11-05

**Authors:** Samar Bizhani, Afsoon Afshari, Ramin Yaghobi, Jamshid Roozbeh

**Affiliations:** 1 Department of Biology, Science and Research Branch, Islamic Azad University, Tehran, Iran; 2 Transplant Research Center, Shiraz University of Medical Sciences, Shiraz, Iran; 3 Nephro-Urology Research Center, Shiraz University of Medical Sciences, Shiraz, Iran

**Keywords:** ceRNA networks, Circular RNA, MicroRNA, BK polyomavirus, Kidney, Transplant

## Abstract

**Background:**

BK polyomavirus (BKPyV) establishes latent human infections, with reactivation linked to the cellular immune response, particularly in kidney transplant recipients (KTRs). Circular RNAs (circRNAs) and microRNAs (miRNAs), classes of noncoding RNAs, are involved in the pathogenesis of kidney diseases and viral infections. CircRNAs act as miRNA “sponges,” diminishing miRNA functions. This study explores the relationship between BKPyV infection and circ-EGLN3 and circ-AKT3, their miRNA targets (miR-1299, miR-296-3p), and their linear counterparts (EGLN3, AKT3), along with miRNA targets (IRF7, CDH1).

**Methods:**

This cross-sectional study included 20 KTRs, divided into 10 BKPyV-infected and 10 non-infected individuals, with an additional control group of 20 healthy individuals. Expression levels of circRNAs, parental genes, and target miRNAs were assessed in blood and urine samples using SYBR Green real-time PCR.

**Results:**

In KTRs with active BKPyV infection, circ-AKT3 and circ-EGLN3 levels were significantly reduced, while their target miRNAs were elevated in both blood and urine compared to non-infected KTRs. ROC curve analysis demonstrated that these circRNAs could significantly differentiate BKPyV-infected individuals from non-infected groups.

**Conclusion:**

This research has established the significance of circular RNAs, particularly circ-AKT3 and circ-EGLN3, and suggests that with additional investigation, these molecules may serve as biomarkers for active BKPyV infection in KTRs, thereby improving the comprehension of BKPyV reactivation.

**Clinical trial number:**

Not applicable.

## Introduction

Kidney transplantation is the optimal therapeutic intervention for end-stage renal disease (ESRD), offering superior patient survival rates and enhanced quality of life compared to dialysis [1]. The advancement of more potent immunosuppressive therapies, coupled with a reduction in the incidence of acute rejection, has led to viral infections becoming a significant factor influencing allograft failure in renal transplantation [2] *BK polyomavirus* (BKPyV), a double-stranded DNA virus, infects over 90% of adults, typically remaining latent in renal tubular and uroepithelial cells [3]. BKPyV, which affects approximately 15% of kidney transplant recipients (KTRs), can lead to BKPyV-associated nephropathy (BKVAN) [4, 5]. Although the virus remains dormant, it can become reactivated when the immune system is weakened, resulting in potentially severe complications that jeopardize graft integrity and patient health. Due to the complexities involved in diagnosing and treating BKPyV-induced toxicity, it is essential to maintain vigilant surveillance and initiate early therapeutic measures for transplant recipients [6].

MicroRNAs (miRNAs), which are classified as small non-coding RNAs, usually span 22 nucleotides in length and are generated from precursor molecules that adopt a hairpin configuration [7] that bind to specific sites within mRNA molecules’ 3′ untranslated region (UTR) [8] to suppress mRNA expression [9]. Recent research indicates that miRNAs are transported across various subcellular compartments to regulate both translation and transcription processes [10].

Circular RNAs (circRNAs) constitute a class of highly stable non-coding RNAs generated via back-splicing events. These molecules exert critical regulatory functions by acting as competitive endogenous RNAs (ceRNAs), sequestering miRNAs and thereby modulating mRNA translation and gene expression networks [11]. Dysregulation of these intricate ceRNA networks contributes to the pathogenesis of kidney diseases [12].

RNA molecules equipped with miRNA response elements (MREs) can operate as ceRNAs, interacting by contending for a collective pool of miRNAs [13]. ceRNA interactions form complex regulatory networks that modulate miRNA activity, introducing a novel and compelling dimension to the miRNA regulatory landscape and giving rise to intricate structures known as competing endogenous RNA networks (ceRNETs). An imbalance within these ceRNETs can trigger the onset and advancement of various diseases [14].

**Fig. 1:**
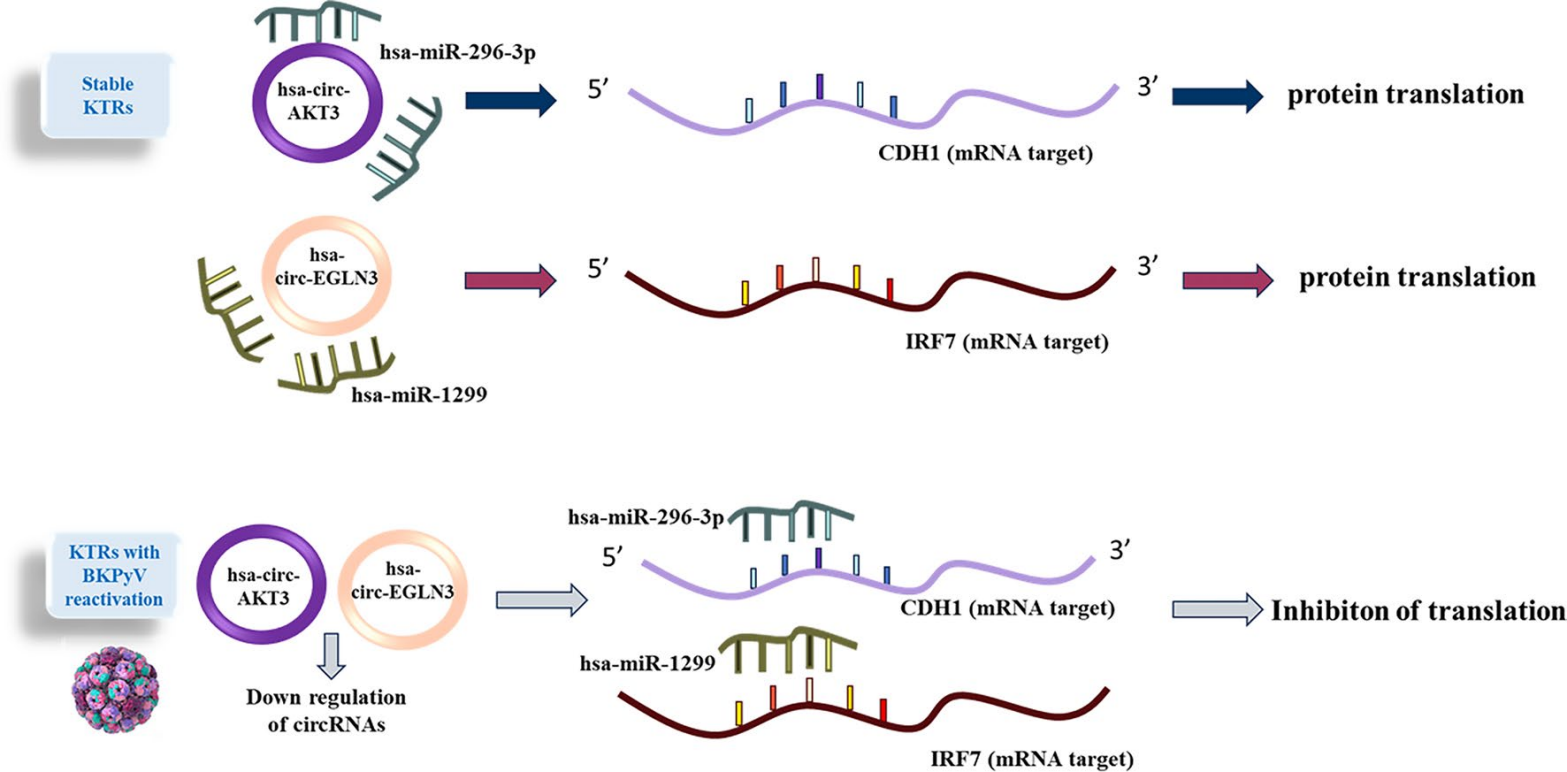
Overview of the study

For instance, circ-AKT3 regulates miR-296-3p/E-cadherin signaling in diabetic nephropathy, while circ-EGLN3 influences miR-1299/IRF7 in renal cell carcinoma (RCC) [15, 16]. Although circ-AKT3 and circ-EGLN3 have not been universally implicated in viral infections, their established roles in modulating key immune and cell-adhesion pathways, such as the miR-296-3p/E-cadherin and miR-1299/IRF7 axes make them compelling candidates for investigating BKPyV pathogenesis. Given that BKPyV tropism depends on epithelial integrity [17] (E-cadherin) and evasion of interferon responses (IRF7) [18], we hypothesize that these circRNAs may influence viral entry, replication, or immune escape mechanisms in renal cells. Therefore, this study aimed to investigate the expression profiles of circ-AKT3, circ-EGLN3, their target miRNAs (miR-296-3p, miR-1299), and genes (CDH1, IRF7) in KTRs with active BKPyV infection to identify potential biomarkers. Furthermore, a schematic figure has been incorporated to provide a clearer overview of the study design and methodology (Fig. 1).

## Materials and methods

### Patient population and demographic data

This cross-sectional study included 20 KTRs admitted to Abu Ali Sina Hospital (Shiraz, Iran) between 2020 and 2022, selected from 350 patients based on BKPyV status confirmed by TaqMan real-time PCR and biopsy (the sample size was determined based on availability). 10 KTRs had active BKPyV infection (BKPyV group), defined by viral load > 10^4^ copies/mL and biopsy-confirmed BKVAN, while 10 had stable grafts with no detectable BKPyV (stable group). Exclusion criteria included age < 18 years, follow-up < 1 year, rejection signs, or sensitization (Luminex flow PRA positive); moreover, those with infections such as adenovirus, CMV, HIV, HBV, and HCV were also excluded. A control group comprising 20 healthy individuals, free from any active infections or inflammatory conditions, was included.

Blood and urine samples were collected under standardized conditions (4 °C, processed within 2 h). Demographic and clinical data are shown in Table 1. RNA concentrations were normalized to total RNA yield per sample.

The Research Ethics Committees of Islamic Azad University- Science and Research Branch and Shiraz University of Medical Sciences granted approval for this study. The methodologies employed adhered to the ethical standards outlined in the Declaration of Helsinki. Informed consent was secured from all participating patients. Donors were sourced from cadavers, selected based on the compatibility of their ABO blood groups.

**Table 1:** Demographic data of KTRs and control group

Group/factor	Control	Stable group%	BKPyV infected group%	*p*-value
Number of participants	20	10	10	-
Gender				> 0.9999
Male	(8) 40%	(7) 70%	(7) 70%	
Female	(12) 60%	(3) 30%	(3) 30%	
Blood type	O+ (10) 50%	O+(3) 30%	O+ (4) 40%	> 0.9999
	B+ (2) 10%	O-(2) 20%	O- (1) 10%	
	O- (6) 30%	A+(1) 10%	A+ (1) 10%	
	A+(2) 10%	B+(3) 30%	B+ (3) 30%	
		AB+(1) 10%	AB+ (1) 10%	
Underlying diseases				0.6667
Diabetes		(1) 10%	(2) 20%	
Hypertension		(9) 90%	(8) 80%	
Age	31.9 ± 6.565	41.4 ± 10.276	50.9 ± 10.082	-

### BKPyV detection

To evaluate BKPyV infection in patients, Taq-Man Real-Time PCR (ABI, First Step Plus, USA) was employed. The extraction of DNA from all collected samples was performed using the Invisorb Spin Virus DNA Blood Mini kit (Invitek, Germany), following the guidelines provided by the manufacturer. In the process of DNA extraction, 200 µl of each plasma sample was designated for analysis. The assessment of the quantity and quality of the extracted DNA was conducted by measuring the optical density at wavelengths of 260 nm and 280 nm, utilizing a NanoDrop spectrophotometer (Thermofisher Scientific, USA). The assessment of BKPyV load in each sample was carried out in alignment with the manufacturer’s instructions, using the BKPyV Taq-Man Real-Time PCR Kit for the real-time PCR analysis (Gensig, Primer Design, UK).

### Isolation of peripheral blood mononuclear cells (PBMCs) and urine sediments

Blood and urine samples were collected from all participants. Blood samples treated with EDTA PBMCs were extracted from the blood samples through the application of Ficoll reagent (Innotrain, Germany). The urine was centrifuged at rcfx10000 for half an hour. Subsequently, the pellet was resuspended in 1 ml of phosphate-buffered saline (PBS). Samples were stored at − 80 °C until RNA extraction.

### RNA extraction and RNase R treatment

Total RNA was extracted from PBMCs and urine sediments using Trizol (Aliyantajhiz, Iran), following the manufacturer’s protocols. RNA integrity was confirmed by 1% agarose gel electrophoresis, and concentration was determined using a NanoDrop spectrophotometer. RNase R treatment (Thermo Fisher scientific) was applied to selectively degrade linear RNAs, enriching circRNAs, as per protocol.

### cDNA synthesis and qPCR

For circRNA and mRNA, 500 ng total RNA was reverse transcribed using EURx cDNA synthesis kit. For miRNA, 50 ng RNA was reverse transcribed using miRNA-specific primers. GAPDH and U6 were used as internal controls for mRNA/circRNA and miRNA, respectively. Primer sequences are listed in Table 2, including housekeeping genes.

qPCR reactions for circRNAs (circ-AKT3 and circ-EGLN3) and mRNAs (CDH1 and IRF7) were performed using SYBR Green Master Mix (Applied Biosystems, USA) in a StepOnePlus Real-Time PCR System (Applied Biosystems). The thermal cycling program was as follows: initial denaturation at 95 °C for 10 min, followed by 40 cycles of 95 °C for 15 s, annealing at primer-specific Tm for 20 s, extension at 72 °C for 30 s. For miRNAs (miR-296-3p and miR-1299), specific stem-loop reverse transcription primers were used. qPCR amplification was performed using the same SYBR Green Master Mix under the following conditions: 2-minute denaturation and polymerase activation step at 95 °C. This was then followed by 40 cycles, which included a 15-second denaturation at 95 °C, and a combined annealing and extension phase at 62 °C for 35 s.

**Table 2:** The sequences of the circular RNAs divergent primers and MiRNAs and mRNAs primers used in the study

circRNAs	circ-AKT3 (hsa_circ_0017252)	F: TCCTTCCAGACAAAAGACCGT R: CGCTCATGATGACTCCCCTC
	circEGLN3 (hsa_circ_0031594)	F: GATCGTAGGAACCCACACGA R: TGATGCAGCGACCATCACC
miRNAs	miR-296-3p	GAGGGTTGGGTGGAGGCTCT
	miR-1299	TTCTGGAATTCTGTGTGAGGGA
	U6	CTCGCTTCGGCAGCACA
mRNAs	AKT3	F: ACCGCACACGTTTCTATGGT R: TGGCCATCTTTGTCCAGCAT
	EGLN3	F: GCCCTCTTACGCAACCAGAT R: AGCACGGTCAGTCTTCAGTG
	CDH1	F: TGGTTCAAGCTGCTGACCTT R: TTAGCCTCGTTCTCAGGCAC
	IRF7	F: GTG AGG GTG TGT CTTCCCTG R: TCG TCA TAG AGGCTGTTG GC
	GAPDH	F: GGACTCATGACCACAGTCCA R: CCAGTAGAGGCAGGGATGAT

CDH1: calcium-dependent glycoprotein named E-cadherin (Gene ID: CDH1); AKT3: adenylate kinase 3; EGLN3: egl-9 family hypoxia-inducible factor 3; IRF7: interferon regulatory factor 7

### Statistical analyses

Statistical analysis was conducted using Graph Pad Prism 5 software for Windows. Normality of data distribution was evaluated using the Kolmogorov–Smirnov test. Since the data did not follow a normal distribution, non-parametric Mann–Whitney U test was applied. The data are expressed as mean ± standard deviation (SD) derived from a minimum of three independent experiments (technical replicates of the PCR assay performed for each patient sample). Thus, for each patient, PCR was conducted in triplicate, and the resulting Ct values were averaged to obtain a single value per patient. All data were compiled using EPSPS version 22 (SPSS, Chicago, IL, USA). The mRNA expression levels for each gene under investigation were determined utilizing the Livak (2^−ΔΔCt^) method. Nonparametric tests were utilized to assess the expression levels in various patient groups. In addition, a two-sided Spearman correlation analysis was carried out to explore the relationships among the variables, using GraphPad Software (Prism 6.01, CA, USA) for this purpose. The determination of the ROC curve, along with the sensitivity and specificity of the analyzed genes, was executed through MedCalc Statistical Software version 17.9 (MedCalc Software, Ostend, Belgium). The criterion for statistical significance was set at *p* < 0.05.

We used bioinformatic tools such as circinteractome, TargetScan, and miRanda algorithms for identifying binding sites for different miRNAs. The mirdb and miRTarBase databases were employed to predict miRNA targets and to validate interactions between microRNAs and their experimentally confirmed targets.

## Results

### circRNAs and linear counterparts’ expression in blood and urine of studied samples

**Fig. 2:**
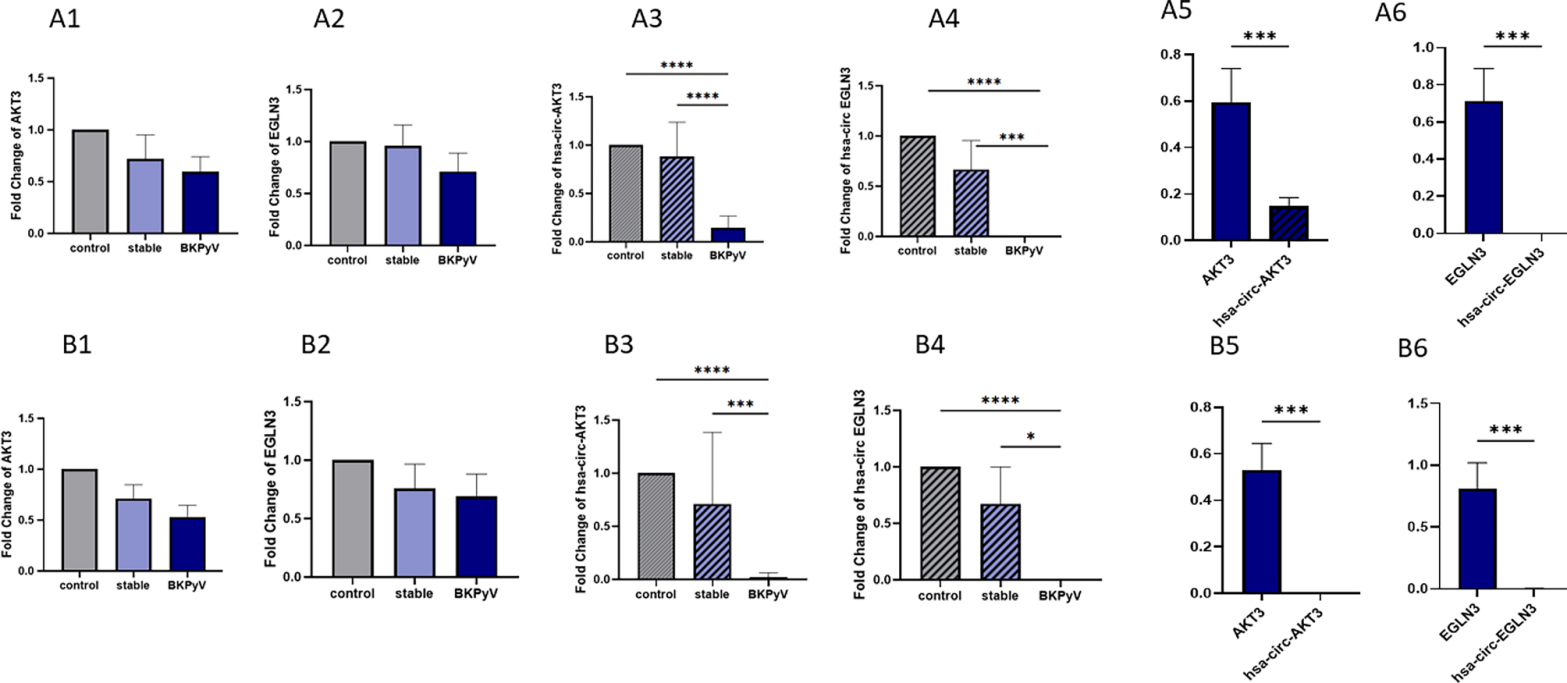
The expression level of studied circular RNAs and their linear counterparts compared in the BKPyV, stable, and control groups in urine (**A**) and blood (**B**) samples **A1**: The expression level of AKT3 in studied groups in urine. **A2**: The expression level of EGLN3 in studied groups in urine. **A3**: The expression level of circ-AKT3 in studied groups in urine. **A4**: The expression level of circ-EGLN3 in studied groups in urine. **B1**: The expression level of AKT3 in studied groups in blood. **B2**: The expression level of EGLN3 in studied groups in blood. **B3**: The expression level of circ-AKT3 in studied groups in blood. **B4**: The expression level of circ-EGLN3 in studied groups in blood (*p < 0.05; **p < 0.01; ***p < 0.001 and ****p < 0.0001)

Figure 2 illustrates the expression profiles of the investigated circular RNAs (circ-AKT3, circ-EGLN3) and their corresponding linear mRNAs (AKT3, EGLN3) in urine (A panels) and blood (B panels) samples from BKPyV-infected KTRs (BKPyV), stable kidney transplant recipients (stable), and healthy controls (control). Comparisons between groups were assessed using the Mann–Whitney U test.

In urine samples, no statistically significant differences were observed in the expression levels of AKT3 (A1) and EGLN3 (A2) among the BKPyV, stable, and control groups. However, circ-AKT3 (A3) expression was significantly reduced in BKPyV patients compared with both control and stable groups (*p* < 0.0001), No significant difference was observed between the stable and control groups. Similarly, circ-EGLN3 (A4) expression was markedly lower in BKPyV patients than in both stable and control groups (*p* < 0.0001), with stable recipients displaying intermediate expression not significantly different from controls.

Direct comparison of linear and circular isoforms revealed a lower proportion of circular to linear transcripts for AKT3 (A5) and EGLN3 (A6) in urine of BKPyV patients (*p* < 0.001). In blood samples, AKT3 (B1) and EGLN3 (B2) linear transcript levels did not differ significantly between groups. In contrast, circ-AKT3 (B3) expression was substantially decreased in BKPyV patients compared with both control and stable groups (*p* < 0.0001), with no notable changes in stable recipients. circ-EGLN3 (B4) was similarly downregulated in BKPyV patients compared with controls (*p* < 0.0001) and stable recipients (*p* < 0.05). Ratios of circular to linear transcripts were significantly lower in BKPyV patients for both AKT3 (B5) and EGLN3 (B6) (*p* < 0.001).

Overall, these results indicate a selective reduction of the circular isoforms of AKT3 and EGLN3 in BKPyV-infected KTRs, detectable in both urine and blood, whereas linear transcripts remained largely unchanged.

### microRNA expression in blood and urine of studied samples

**Fig. 3:**
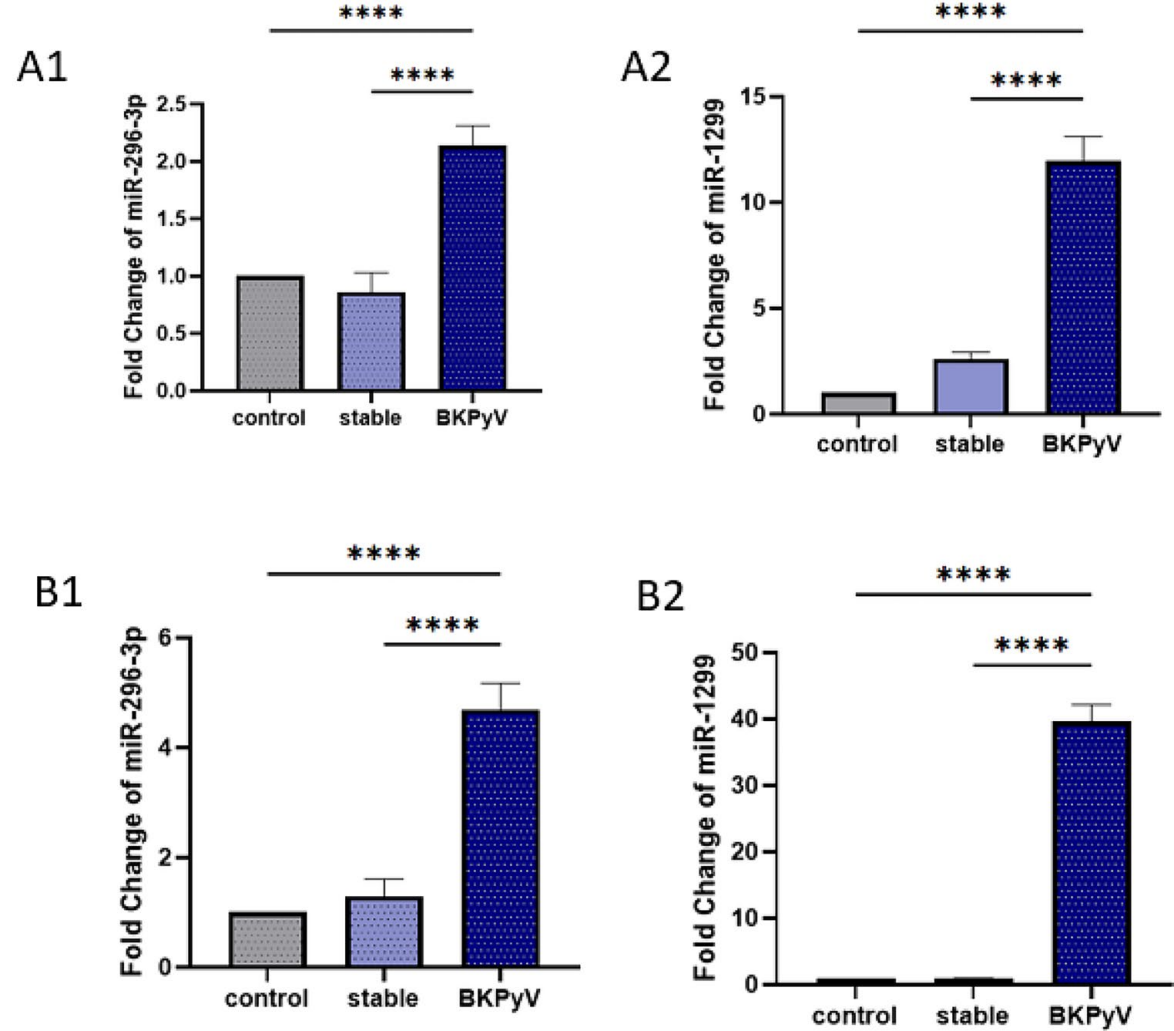
The expression level of studied microRNAs and compared in the BKPyV, stable, and control groups in urine (**A**) and blood (**B**) samples. **A1**: The expression level of miR-296-3p in studied groups in urine. **A2**: The expression level of miR-1299 in studied groups in urine. **B1**: The expression level of miR-296-3p in studied groups in blood. **B2**: The expression level of miR-1299 in studied groups in blood (*p < 0.05; **p < 0.01; ***p < 0.001 and ****p < 0.0001)

Figure 3 presents the expression levels of the investigated microRNAs (miR-296-3p and miR-1299) in urine (A panels) and blood (B panels) from BKPyV-infected KTRs (BKPyV), stable KTRs recipients (stable), and healthy controls (control). Group differences were analyzed using the non-parametric Mann–Whitney U test.

In urine samples, the expression level of miR-296-3p (A1) was significantly higher in BKPyV-infected kidney transplant recipients compared with both stable recipients and healthy controls (*p* < 0.0001). Similarly, miR-1299 (A2) expression was markedly elevated in BKPyV patients compared with both stable recipients and controls (*p* < 0.0001). In blood samples, miR-296-3p (B1) was significantly upregulated in BKPyV patients compared with stable and control groups (*p* < 0.0001). The expression of miR-1299 (B2) followed a similar pattern, showing a substantial increase in BKPyV patients compared with both stable recipients and controls (*p* < 0.0001). Overall, these data indicate consistent and robust upregulation of both miR-296-3p and miR-1299 in BKPyV-infected patients across urine and blood, suggesting that changes in these miRNAs may be associated with BKPyV infection status in KTRs.

### Target gene expression in blood and urine of studied samples

**Fig. 4:**
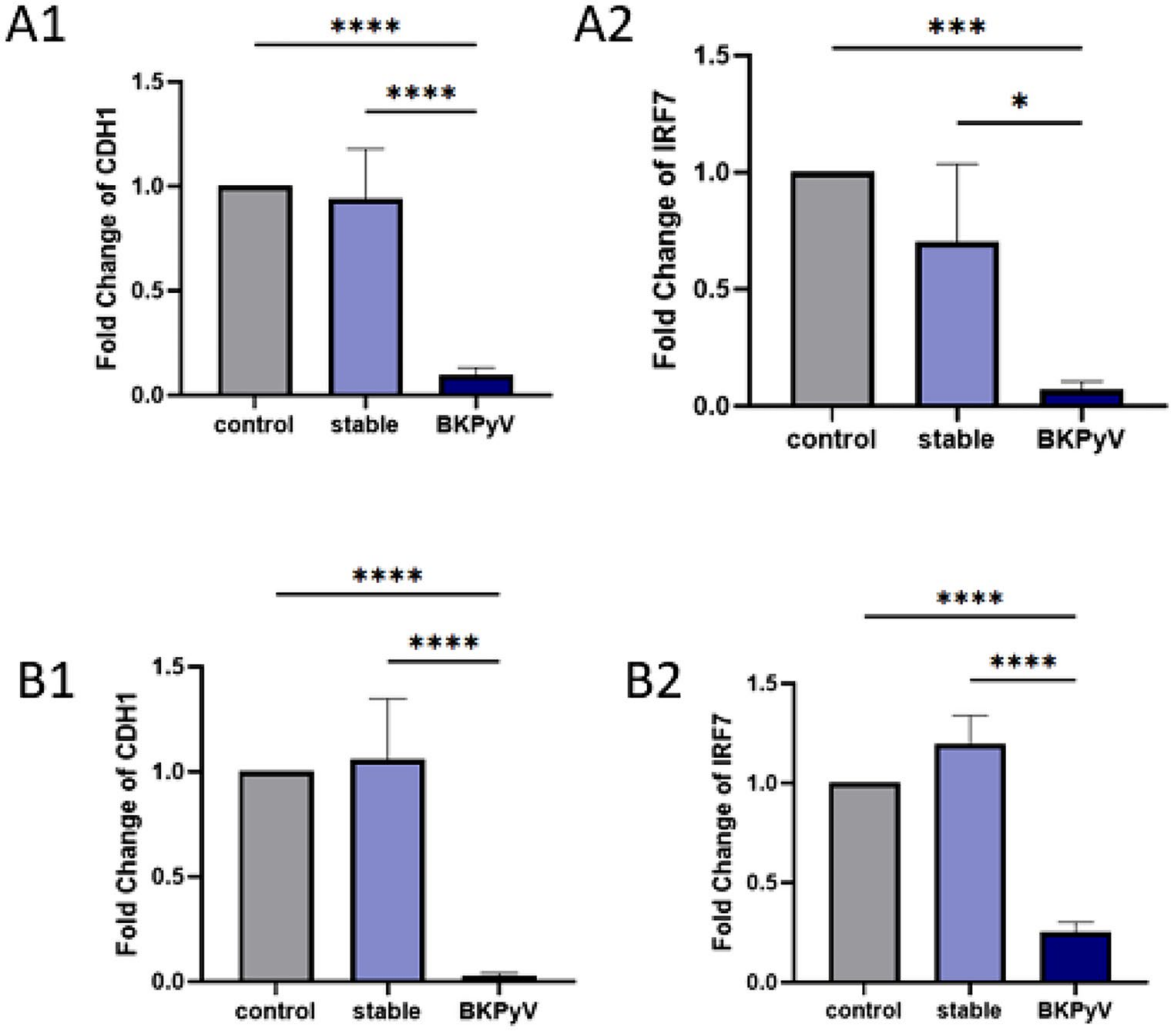
The expression level of studied target genes in the BKPyV, stable, and control groups in urine (**A**) and blood (**B**). **A1**: The expression level of CDH1 in studied groups in urine. **A2**: The expression level of IRF7 in studied groups in urine. **B1**: The expression level of CDH1 in studied groups in blood. **B2**: The expression level of IRF7 in studied groups in blood (*p < 0.05; **p < 0.01; ***p < 0.001 and ****p < 0.0001)

Figure 4 shows the expression levels of the mRNA targets of miR-296-3p (CDH1) and miR-1299 (IRF7) in urine (A panels) and blood (B panels) samples from BKPyV-infected kidney transplant recipients (BKPyV), stable kidney transplant recipients (stable), and healthy controls (control). Statistical significance was determined using the Mann–Whitney U test for pairwise comparisons.

In urine, CDH1 expression (A1) was markedly reduced in the BKPyV group compared with both control and stable groups (*p* < 0.0001 for both comparisons). IRF7 (A2) also showed a significant decrease in BKPyV patients relative to controls (*p* < 0.001) and stable recipients (*p* < 0.05).

In blood, CDH1 expression (B1) was significantly downregulated in the BKPyV group compared with both control and stable groups (*p* < 0.0001). Similarly, IRF7 expression (B2) was markedly lower in BKPyV patients compared with both other groups (*p* < 0.0001 for both comparisons).

### The receiver operating characteristic (ROC) curve analysis of expression level of studied circular RNAs in urine and blood

**Fig. 5:**
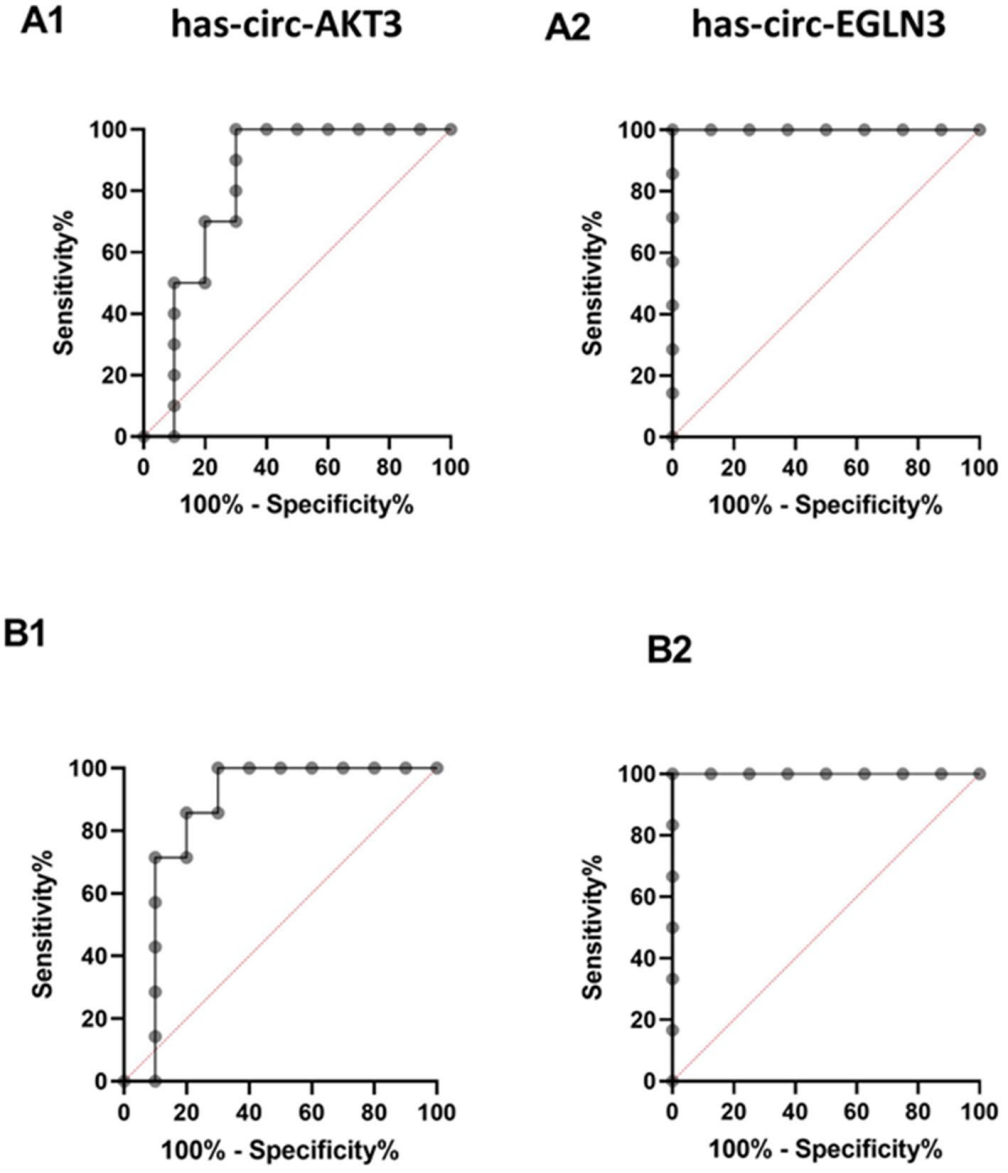
The ROC curve of circular RNAs in the BKPyV and stable groups in urine (**A**) and blood (**B**) samples

Figure 5 presents the receiver operating characteristic (ROC) curves for the evaluated circular RNAs, illustrating their diagnostic performance across a range of classification thresholds. The area under the ROC curve (AUC) was employed as a quantitative indicator of overall discriminatory capacity. The AUC, sensitivity, specificity, and optimal cutoff values were obtained from Youden’s index.

For hsa-circ-EGLN3, ROC analysis revealed exceptional diagnostic accuracy in both blood and urine specimens. In blood, hsa-circ-EGLN3 achieved perfect discrimination, with an AUC of 1.00 (*p* = 0.0019) at a cutoff value of 0.01, corresponding to 100% sensitivity, 100% specificity, and predictive values of 100% for both PPV and NPV. Similarly, in urine, the biomarker maintained an AUC of 1.00 (*p* = 0.0012) at a cutoff value of 0.000045, again yielding 100% sensitivity, 100% specificity, and PPV and NPV of 100%.

In contrast, the diagnostic performance of hsa-circ-AKT3 was robust but comparatively less consistent. In blood, it demonstrated an AUC of 0.8571 (*p* = 0.0147) at a cutoff value of 0.02, with sensitivity of 85.71%, specificity of 80%, PPV of 81.08%, and NPV of 84.85%. In urine, the AUC was 0.83 (*p* = 0.0156) at a cutoff value of 0.5, achieving 100% sensitivity, 70% specificity, PPV of 76.9%, and NPV of 100%.

### Comparison of the expression and correlation of circRNAs and their target miRNAs

**Fig. 6:**
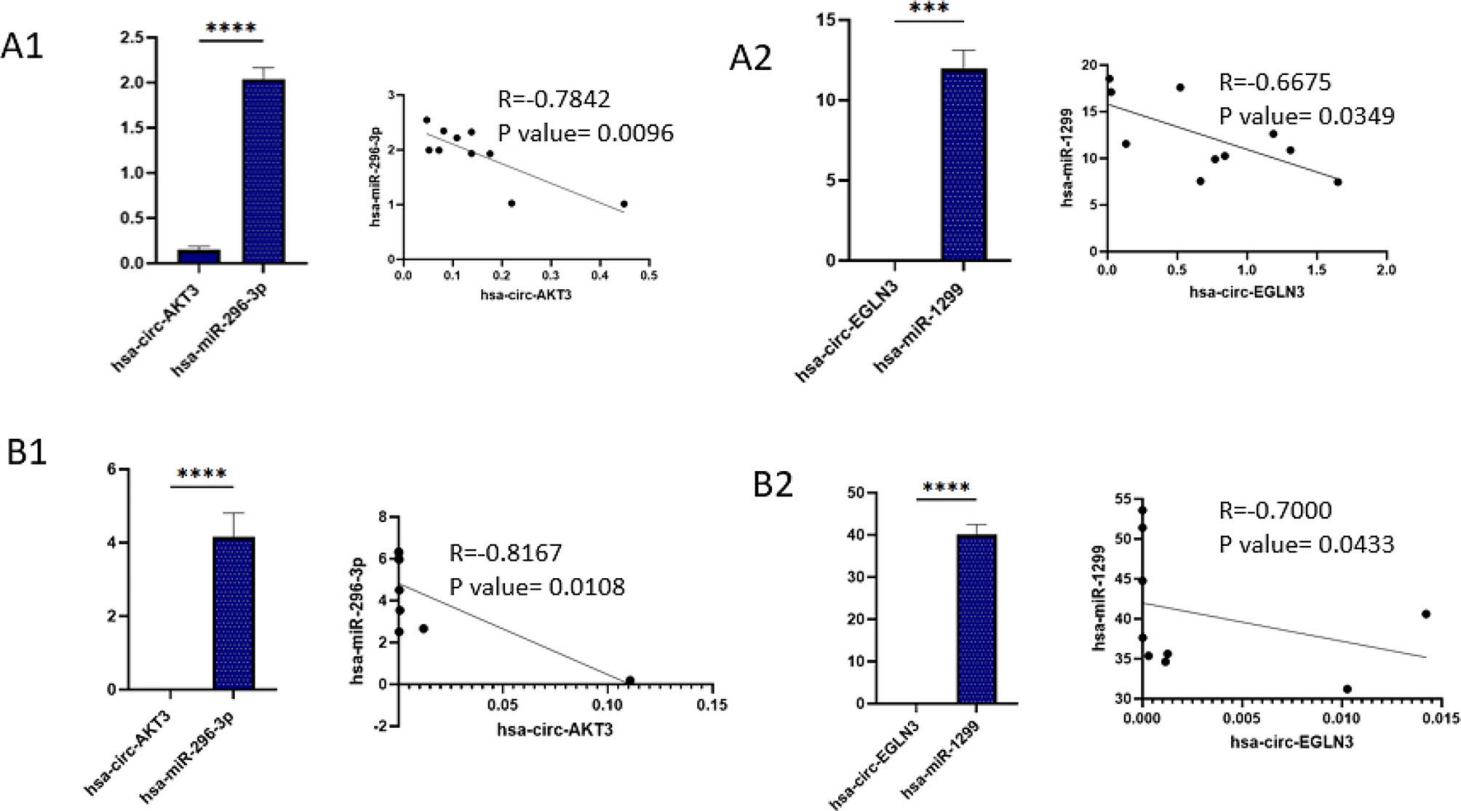
Comparison of the expression of circular RNAs and their target microRNAs and their correlation in the BKPyV infected groups in urine (**A**) and blood (**B**) samples

Figure 6 shows the correlation analysis between the expression levels of circRNAs and their associated miRNAs in both urine and blood samples of kidney transplant recipients. Correlation analyses were performed using Spearman’s test.

A strong and statistically significant negative correlation was observed between circ-AKT3 and miR-296-3p in both urine and blood. Similarly, circ-EGLN3 expression was inversely correlated with miR-1299 levels. These findings support the hypothesis that decreased circRNA expression may lead to the upregulation of their complementary miRNAs, consistent with the proposed ceRNA regulatory mechanism in BKPyV infection.

### Comparison of the expression and correlation of miRNAs and their target genes

**Fig. 7:**
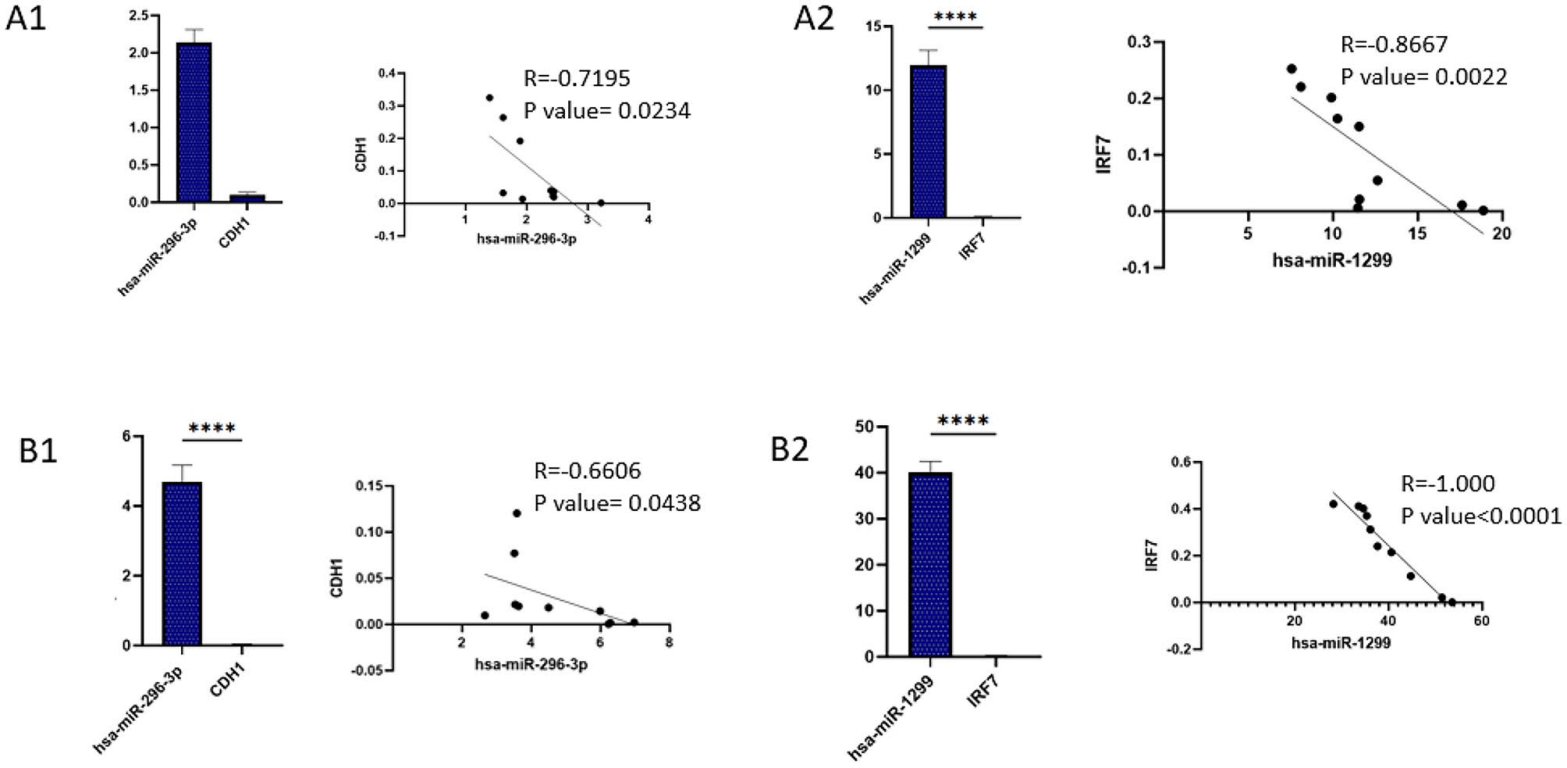
Comparison of the expression of microRNAs and their target genes and their correlation in the BKPyV infected groups in urine (**A**) and blood (**B**) samples

Figure 7 presents the correlation analysis between the studied miRNAs and their mRNA targets in urine and blood samples. Non-parametric Spearman correlation was applied to assess associations between variables.

miR-296-3p showed a significant inverse correlation with CDH1 mRNA levels. Likewise, miR-1299 expression was inversely correlated with IRF7 mRNA levels in both sample types. These results may confirm the post-transcriptional regulatory effect of these miRNAs on their mRNA targets, suggesting that miRNA upregulation contributes to the downregulation of key mRNAs during BKPyV infection.

## Discussion

In this study, we thoroughly examined the expression profiles of two circular RNAs (circ-AKT3 and circ-EGLN3), their related miRNAs (miR-296-3p and miR-1299), and target mRNAs (CDH1 and IRF7) in both urine and blood samples from KTRs with or without BKPyV infection. To our knowledge, this is the first study to identify a circRNA–miRNA–mRNA regulatory axis in BKPyV infection.

We observed a significant decrease in circ-AKT3 and circ-EGLN3 in both urine and blood samples from BKPyV-infected KTRs compared with stable recipients and healthy controls, while the levels of their linear mRNA counterparts remained largely unchanged. This selective reduction suggests that BKPyV infection may specifically affect circRNAs rather than broadly impacting the transcription of their host genes. CircRNAs are generally stable because of their covalently closed structure [19] and have been linked to viral pathogenesis, including roles in immune modulation and host virus interactions [20] In parallel, miR-296-3p and miR-1299 were significantly elevated in both blood and urine of BKPyV-infected recipients. The inverse correlation between each circRNA and its corresponding miRNA supports the ceRNA hypothesis, wherein circRNAs act as miRNA sponges, limiting miRNA availability to bind target mRNAs [21]. Reduced circRNA abundance in BKPyV infection would therefore enhance miRNA activity, contributing to repression of target genes. The mRNA targets of these miRNAs, CDH1 and IRF7 were significantly downregulated in BKPyV-infected patients. CDH1 encodes E-cadherin, essential for epithelial integrity [22]; its loss may facilitate viral spread within the urinary tract. IRF7 acts as a master controller of type I interferon responses [23], and its suppression may diminish antiviral immunity. The functional links identified imply that the circ-AKT3/miR-296-3p/E-cadherin and circEGLN/miR-1299/IRF7 axes may have direct impact on BKPyV pathogenesis by simultaneously compromising physical barriers and reducing immune defenses. Previous analyses of various viral infections, such as HBV, EBV, and cytomegalovirus (CMV), have shown that the virus can induce dysregulation of host circRNAs [20, 24]. Likewise, elevated miR-296-3p and miR-1299 levels have been reported in inflammatory conditions [25, 26] and renal carcinoma, where they were implicated in immune evasion and tumor progression [16, 27]. Our integrated data support a mechanistic model in which BKPyV infection disrupts circRNA expression potentially through interference with host splicing machinery or RNA decay pathways resulting in decreased levels of circ-AKT3 and circ-EGLN3. This downregulation reduces miRNA sequestration, allowing miR-296-3p and miR-1299 to accumulate and exert stronger post-transcriptional repression of CDH1 and IRF7. The resulting loss of epithelial adhesion molecules and antiviral signaling factors could create a permissive environment for viral persistence and propagation. The limitations of this study include a moderate sample size and the lack of in vitro or in vivo mechanistic validation. To establish the diagnostic performance and ascertain whether the identified RNA signatures pertain specifically to BKPyV or can be generalized to other viral infections post-transplant, larger multicenter studies are necessary. Experimental validation, such as luciferase reporter assays and knockdown/overexpression models, will be essential to directly confirm the predicted ceRNA interactions.

One of the primary limitations of this study is the relatively small sample size, with 10 patients in each KTR group and 20 healthy controls. While the findings provide preliminary insights into the potential role of circ-AKT3 and circ-EGLN3 as biomarkers for BKPyV infection, the limited number of participants reduces the statistical power and generalizability of the results. Consequently, these observations should be interpreted with caution. Larger, multicenter studies are warranted to validate these findings and to establish the robustness of the proposed circRNA–miRNA–mRNA regulatory axis in BKPyV pathogenesis.

## Conclusions

We suggest a potential circRNA–miRNA–mRNA regulatory axis in BKPyV infection, involving the downregulation of circ-AKT3 and circ-EGLN3, upregulation of miR-296-3p and miR-1299, and associated repression of CDH1 and IRF7 genes. Such molecular alterations could contribute to BKPyV pathogenesis by impairing epithelial integrity and modulating antiviral immune responses. Moreover, components of this axis may hold promise as non-invasive diagnostic biomarkers. These observations provide further insight into host–virus interactions in kidney transplantation and may guide future efforts toward biomarker development and targeted therapeutic strategies.

## Data Availability

All data generated or analyzed during this study are included in this published article.

## Abbreviations

BKPyV: BK polyomavirus
KTRs: Kidney transplant recipients
circRNAs: circular RNAs
miRNAs: microRNAs
ESRD: End-stage renal disease
BKVAN: BKPyV nephropathy
NK: Natural Killer
DCs: Dendritic cells
UTR: Untranslated region
RCC: Renal cell carcinoma
MREs: miRNA response elements
ceRNAs: Competing endogenous RNAs
ceRNETs: ceRNA networks
CMV: Cytomegalovirus
HBV: Hepatitis B
HCV: Hepatitis C
HIV: Human immunodeficiency virus (HIV)
Cr: Creatinine (Cr)
CDH1: Calcium-dependent glycoprotein
AKT3: Adenylate kinase 3
EGLN3: Egl-9 family hypoxia-inducible factor 3
IRF7: Interferon regulatory factor 7
ROC: Receiver operating characteristic
AUC: Area Under the ROC Curve
